# Neural Abnormalities in Panic Disorder and Agoraphobia: A Meta-Analysis of Functional Activation Studies

**DOI:** 10.1192/j.eurpsy.2024.140

**Published:** 2024-08-27

**Authors:** C. Baten, A. M. Klassen, G. Zamora, J. H. Shepherd, A. Badawia, A. Kailay, C. R. Leung, J. Sahota, S. Saravia, J. A. Miller, P. Hamilton, M. D. Sacchet, I. H. Gotlib, E. Woo, D. W. Hedges, C. H. Miller

**Affiliations:** ^1^Department of Psychology, California State University, Fresno, Fresno; ^2^Department of Psychology, Palo Alto University, Palo Alto, United States; ^3^Department of Biological and Medical Psychology, University of Bergen, Bergen, Norway; ^4^Department of Psychiatry, Massachusetts General Hospital, Harvard Medical School, Boston; ^5^Department of Psychology, Stanford University, Palo Alto; ^6^Department of Psychology, Brigham Young University, Provo, United States

## Abstract

**Introduction:**

Panic disorder (PD) and agoraphobia (AG) are highly comorbid anxiety disorders with an increasing prevalence that have a significant clinical and public health impact but are not adequately recognized and treated. Although the current functional neuroimaging literature has documented a range of neural abnormalities in these disorders, primary studies are often not sufficiently powered and their findings have been inconsistent.

**Objectives:**

This meta-analysis aims to advance our understanding of the neural underpinnings of PD and AG by identifying the most robust patterns of differential neural activation that differentiate individuals diagnosed with one of or both these disorders from age-matched healthy controls.

**Methods:**

We conducted a comprehensive literature search in the PubMed database for all peer-reviewed, whole-brain, task-based functional magnetic resonance imaging (fMRI) activation studies that compared adults diagnosed with PD and/or AG with age-matched healthy controls. Each of these articles was screened by two independent coding teams using formal inclusion criteria and according to current PRISMA guidelines. We then performed a voxelwise, whole-brain, meta-analytic comparison of PD/AG participants with age-matched healthy controls using multilevel kernel density analysis (MKDA) with ensemble thresholding (p<0.05-0.0001) to minimize cluster size detection bias and 10,000 Monte Carlo simulations to correct for multiple comparisons.

**Results:**

With data from 34 primary studies and a substantial sample size (N=2138), PD/AG participants, relative to age-matched healthy controls, exhibited a reliable pattern of statistically significant, (p<.05-0.0001; FWE-corrected) abnormal neural activation in multiple brain regions of the cerebral cortex and basal ganglia across a variety of experimental tasks.

**Conclusions:**

In this meta-analysis we found robust patterns of differential neural activation in participants diagnosed with PD/AG relative to age-matched healthy controls. These findings advance our understanding of the neural underpinnings of PD and AG and inform the development of brain-based clinical interventions such as non-invasive brain stimulation (NIBS) and treatment prediction and matching algorithms. Future studies should also investigate the neural similarities and differences between PD and AG to increase our understanding of possible differences in their etiology, diagnosis, and treatment.

**Disclosure of Interest:**

None Declared

